# Supplementary material for: A FAK/HDAC5 signaling axis controls osteocyte mechanotransduction
Source: Nat Commun. 2020 Jul 1;11:3282. doi: 10.1038/s41467-020-17099-3 (PMC7329900; doi:10.1038/s41467-020-17099-3)
Supplement: Supplementary file 1 — Supplementary Information [file 41467_2020_17099_MOESM1_ESM.pdf]

Supplemental Figure 1

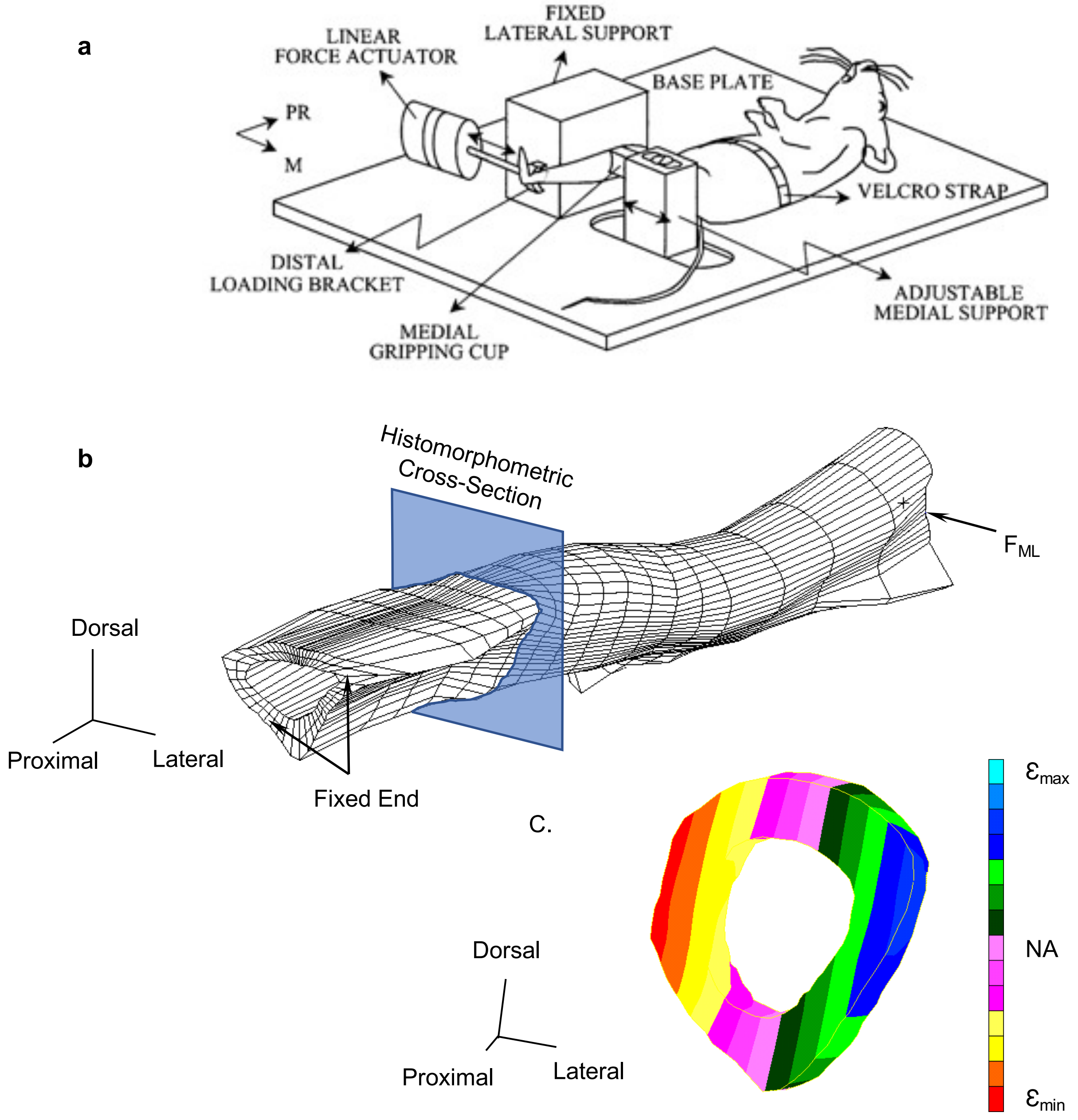

Supplemental Figure 1

(a) Schematic of cantilever bending loading device illustrating mechanical stimulation of the right tibia via a computer-controlled actuator that applies load to the lateral surface of the distal tibia (with permission from (93)). (b) MicroCT based finite element model of mouse tibia used to quantify normal strain distribution induced when the tibia is placed in cantilever bending via end loading (FML, per (93)). The FE mesh is aligned opposite of (a), with the fixed proximal end of the tibia to the left in order to visualize the application of force to the distal tibia. (c) Normal strain distribution was calculated in the tibia mid-shaft at the site of histomorphometric analysis (2.5 mm proximal to tibia-fibula junction). The induced bending environment places the lateral cortex in compression (emax), the medial cortex in tension (emin), with the neutral axis noted in pink (i.e., 0 normal strain). This orientation of the neutral axis acting on the tibia midshaft, although achieved via exogenous loading in this study, closely resembles that induced during free ambulation (1, 2).

Supplemental Figure 2 (related to Figure 1)

a **Micro CT analyses**

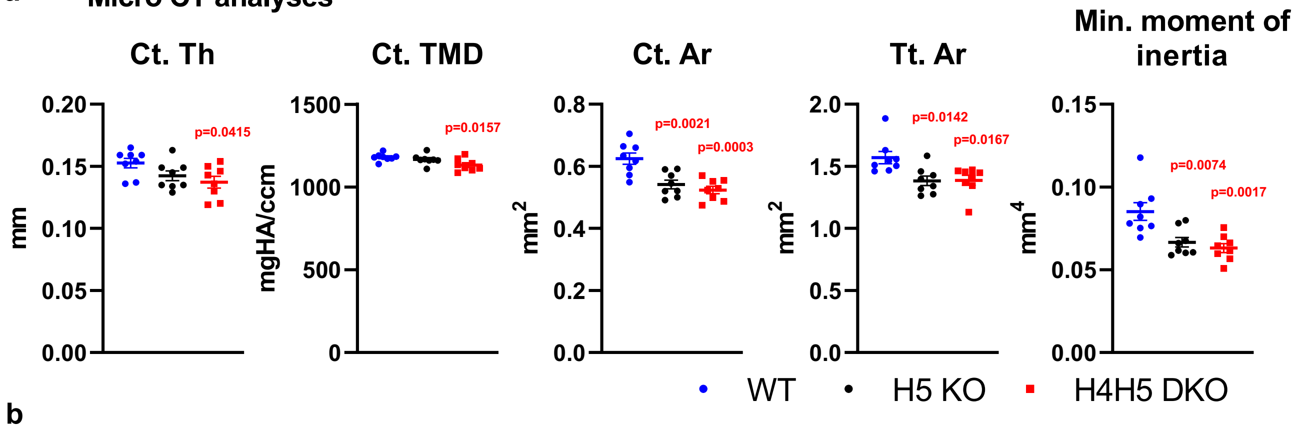

b **Three-point bending test**

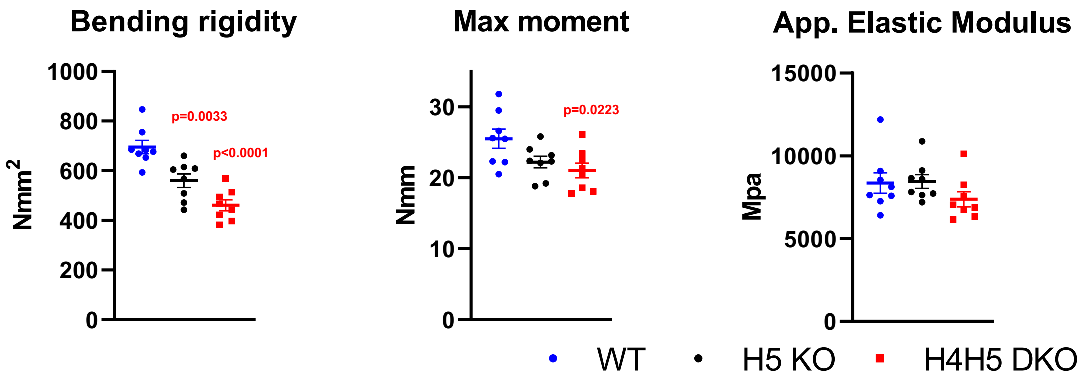

c

Bending rigidity versus bone mass

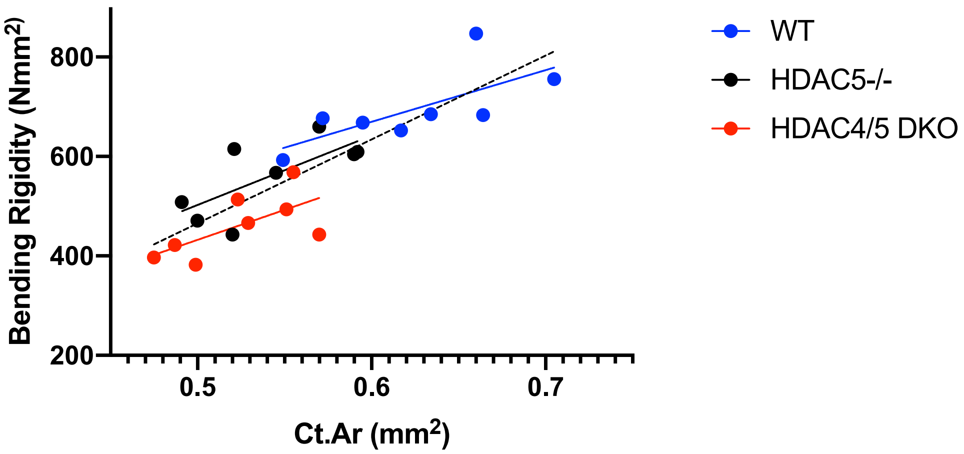

Supplemental Figure 2

(a) Femur midshaft microCT results comparing 8 week old wildtype (WT), HDAC5 global knockout (H5KO; HDAC5-/-, HDAC4fl/fl), and HDAC5 and HDAC4 double knockout (H4H5 DKO; HDAC5-/-, HDAC4 fl/fl, DMP1-Cre) (n=8/group). As previously reported (3), Hdac4/5DKO animals have smaller bones with cortical osteopenia. Micro-CT and mechanical testing were not performed for Hdac4 single knockout mice as we previously reported that these animals show normal cortical bone mass and normal cortical bone histology (3). (b) Three-point bend tests were then performed on the femoral diaphysis from the same animals as used for microCT revealing lower bending rigidity and maximum moment in the Hdac4/5DKO, but no differences in the apparent elastic modulus, suggests that the observed differences in mechanical properties are likely a result of differences in the bone geometry and not differences in the intrinsic material properties of the cortical bone. (c) We plotted bending rigidity (measured by 3 point testing) versus cortical area (measured by micro-CT) for all mice in the study. Notably, the relationship between bending rigidity and bone strength was similar in all groups (e.g., no significant difference in slope) suggesting that the mutant mice did not have deficits in this measure of bone quality, consistent with the findings for apparent elastic modulus above. (n=8/group) p values adjusted for multiple comparisons vs WT are shown (a-c). One-way ANOVA followed by Tukey-Kramer post hoc test (a) was used. Data are expressed as mean ± SEM. Source data are provided as a Source Data file.

Supplemental Figure 3 (related to Figure 2)

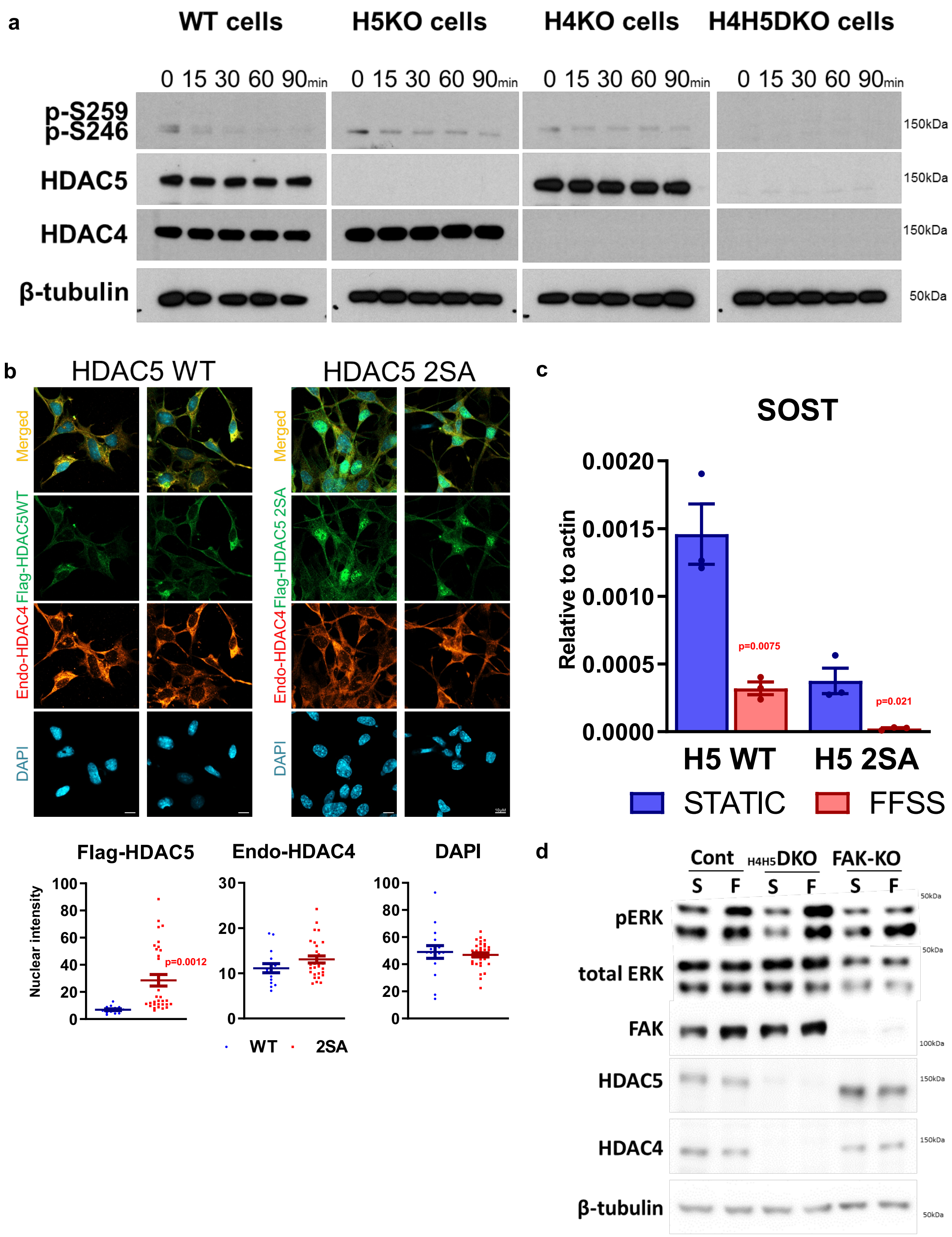

Supplemental Figure 3

(a) Ocy454 cells of the indicated “genotype” were treated with FFSS for the indicated time followed by immunoblotting. HDAC4/5 serine phosphorylation at S246 (HDAC4) and S259 (HDAC5) was mildly reduced by FFSS. (b) Immunocytochemistry in HDAC5-deficient Ocy454 cells transduced with lentiviral constructs expressing FLAG-tagged wild type (WT) HDAC5 and HDAC5 S259/498A (2SA) was performed and then analyzed by confocal microscopy HDAC5 2SA mutant shows constitutive nuclear localization. N=15 WT, n=30 2SA. P vs WT. (c) HDAC5-deficient Ocy454 cells expressing the indicated HDAC5 variant were treated with FFSS followed by RNA isolation and RT-qPCR for Sost. While basal Sost expression is reduced in HDAC5 2SA cells, levels are reduced further in response to FFSS. P vs STATIC, n=3. (d) Control, HDAC4/5-deficient, and FAK-deficient Ocy454 cells were treated plus/minus FFSS (S = static, F = FFSS) for 10 minutes followed by immunoblotting as indicated. HDAC4/5-deficient cells show intact FFSS-induced p42/44 ERK phosphorylation. FAK-deficient cells show preserved FFSS-induced p42 ERK phosphorylation. Two-sided unpaired t-test was used (b, c). Data are expressed as mean ± SEM. Each experiment was repeated three times (a, d). Source data are provided as a Source Data file.

Supplemental Figure 4 (related to Figure 3)

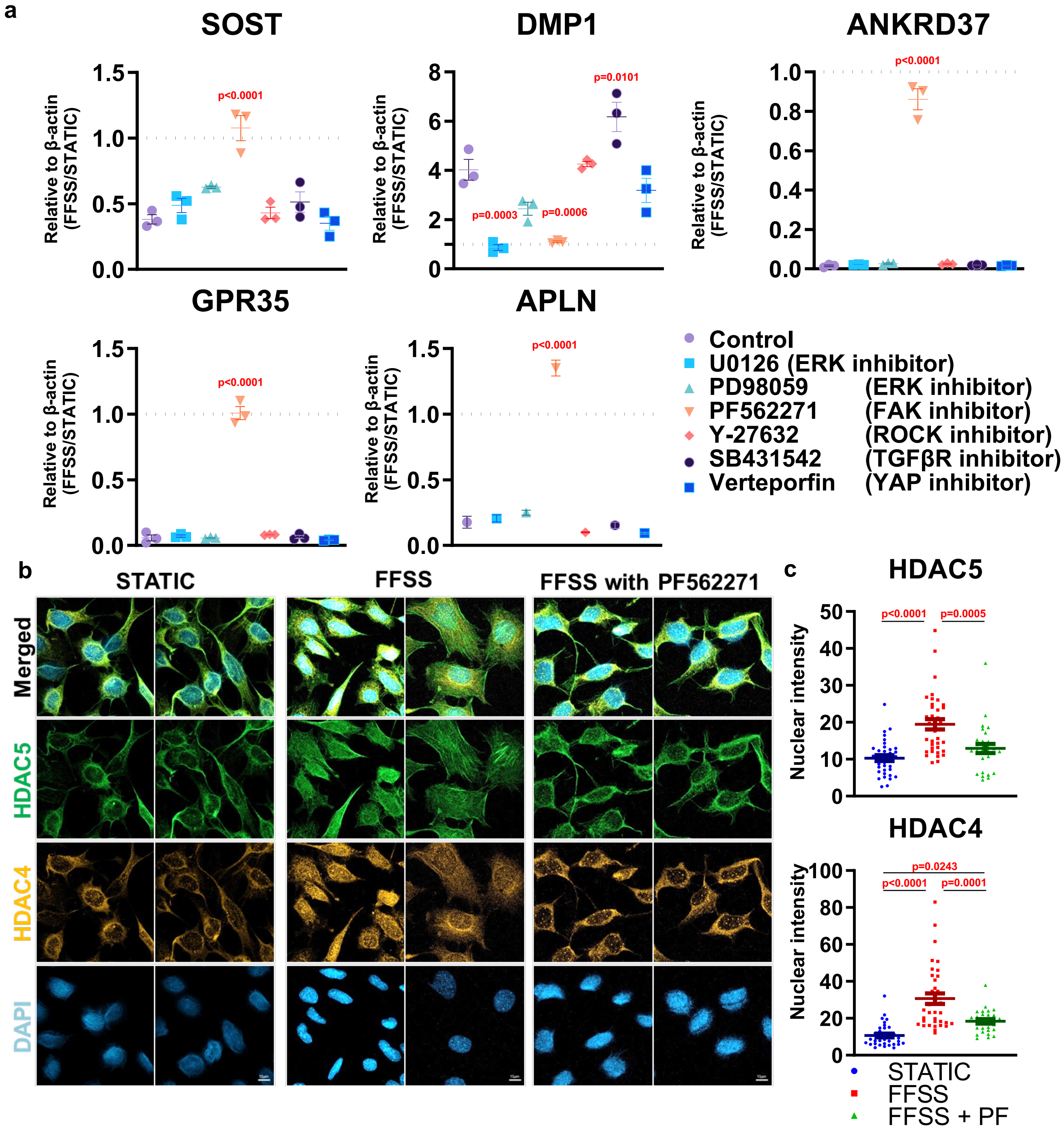

Supplemental Figure 4

(a) Ocy454 cells were pre-treated for 1 hour with small molecule inhibitors of candidate mechanosensitive signaling pathways (U0126, PD98059, PF562271, y-27632, SB431542, Verteporfin) followed by 3-hour FFSS treatment. Subsequently, RNA was isolated for RT-qPCR. Data are shown as the ratio between gene expression in FFSS versus static conditions for each inhibitor pretreatment. U0126; ERK inhibitor, PD98059; ERK inhibitor, PF562271; FAK inhibitor, y-27632; Rock inhibitor, SB431542; TGFßR inhibitor, Verteporfin; YAP. Only FAK inhibitor pre-treatment blocks the reduction of gene expression by FFSS. One-way ANOVA (Tukey-Kramer) was performed. p values adjusted for multiple comparisons vs control are shown, n=3/group. (b) HDAC4/5 immunocytochemistry was performed followed by confocal microscopy. One hour FAK inhibitor (PF562271) pre-treatment blocked FFSS induced HDAC5 and HDAC4 translocation from cytosol compartment to nuclei. Pictures show two representative images of each staining. HDAC5 (green), HDAC4 (red), and DAPI (blue). Each experiment was repeated three times. (c) The densitometric intensity of HDAC5 and HDAC4 was measured by ImageJ. Multiplicity adjusted p are used. N=36/group, STATIC and FFSS. N=28, FFSS + PF. One-way ANOVA followed by Tukey-Kramer post hoc test (a, c) was used. Data are expressed as mean ± SEM. Source data are provided as a Source Data file.

Supplemental Figure 5 (related to Figure 3)

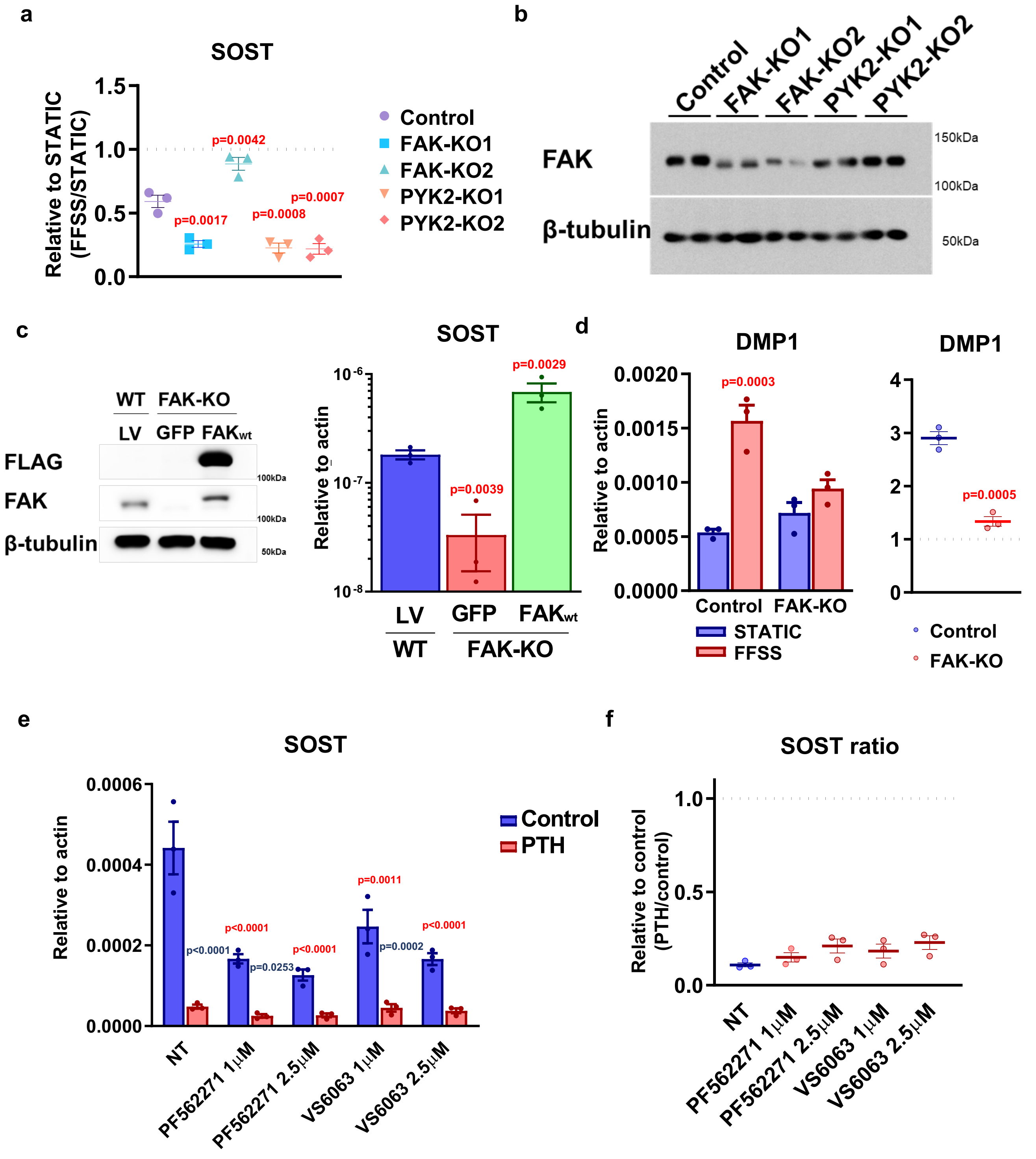

Supplemental Figure 5

Cas9-expressing Ocy454 cells were infected with lentiviruses expressing sgRNAs targeting FAK or the related kinase PYK2. An empty vector "lentiguide" (LG-EV) was used as a control. LV-infected cells were selected by antibiotic resistance, and bulk cell populations were treated plus/minus FFSS followed by Sost RT-qPCR. FAK-KO population 2 showed blunted FFSS-induced Sost suppression. (n=3) p vs control (a), and reduced overall FAK protein levels (b). (c) FAK-deficient single cell clone 1-4 was infected with the indicated lentiviral construct expressing GFP or FAK cDNA. Cells were grown at 37ºC for 14 days followed by immunoblotting (left) and RT-qPCR (right). FAK-deficient cells show low basal SOST expression which is rescued with reintroduction of FAK. (d) Control and single cell FAK mutant cells were subjected to FFSS and then Dmp1 mRNA levels were measured by RT-qPCR. FFSS-induced Dmp1 up-regulation does not occur in FAK mutant cells. (e, f) Ocy454 cells were treated plus/minus PTH (242 nM) and the indicated doses of FAK inhibitors. FAK inhibitor treatment does not block PTH-induced Sost suppression. Panel E shows Sost mRNA levels for each treatment (n=3). P (red) vs control-NT. P (blue) vs each control. Panel F shows the ratio of PTH-treatment versus vehicle in the presence of each dose of FAK inhibitors. PTH treatment further suppresses Sost expression even in the presence of FAK inhibitor treatment. Two-sided unpaired t-test (c, d in the left panel) and one-way ANOVA followed by Tukey-Kramer post hoc test (a, d in the left panel, e, f) were used. Multiplicity adjusted p values are used (a, c in the right panel, d in the left panel, e). Data are expressed as mean ± SEM. Source data are provided as a Source Data file.

Supplemental Figure 6 (related to Figures 3 and 6)

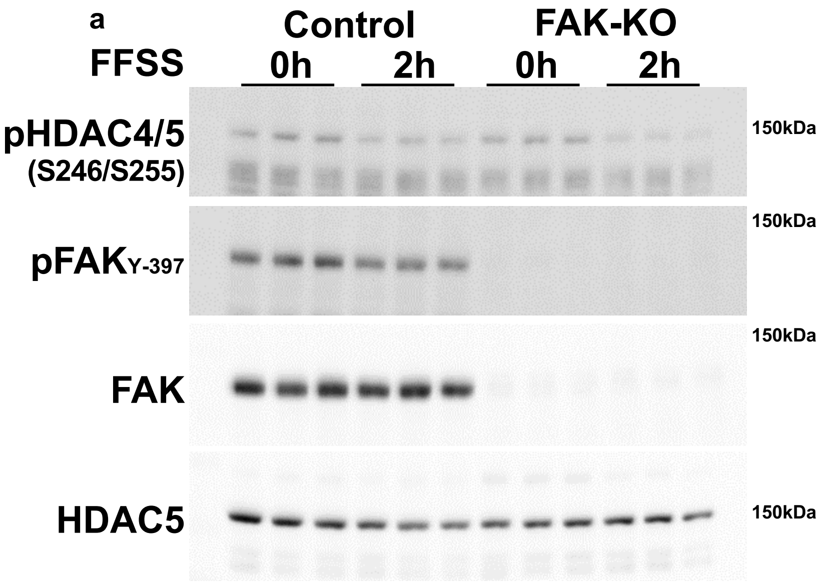

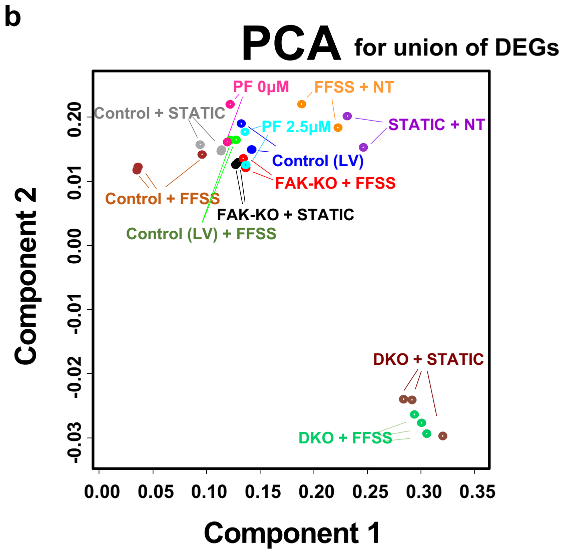

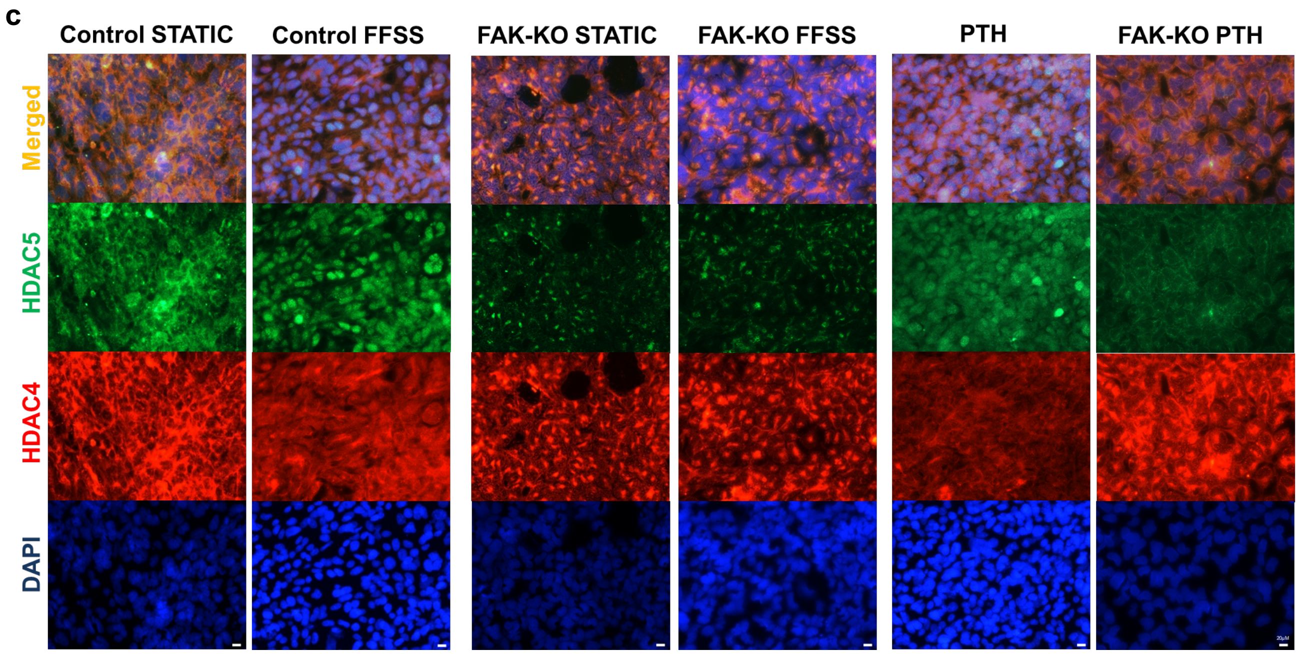

Supplemental Figure 6

(a) FAK deficient cells (single cell clone 1-4) were treated with FFSS as indicated followed by immunoblotting for HDAC4/5 at N-terminal phosphorylation sites. Both control and FAK-mutant cells show intact (mild) FFSS-induced reductions in HDAC4/5 serine phosphorylation. (b) Principal component analysis for all 28 RNA-seq libraries analyzed in this manuscript. Here, the union of all differentially expressed genes was used to generate this plot. (c) Immunocytochemistry was performed in control (pLentiGuide-empty vector, LG-EV) or FAK-mutant cells followed by confocal microscopy. Abnormal HDAC4/5 localization is noted in FAK mutant cells, and FAK mutant cells fail to show FFSS-induced HDAC4/5 nuclear translocation. Source data are provided as a Source Data file.

Supplemental Figure 7 (related to Figure 6)

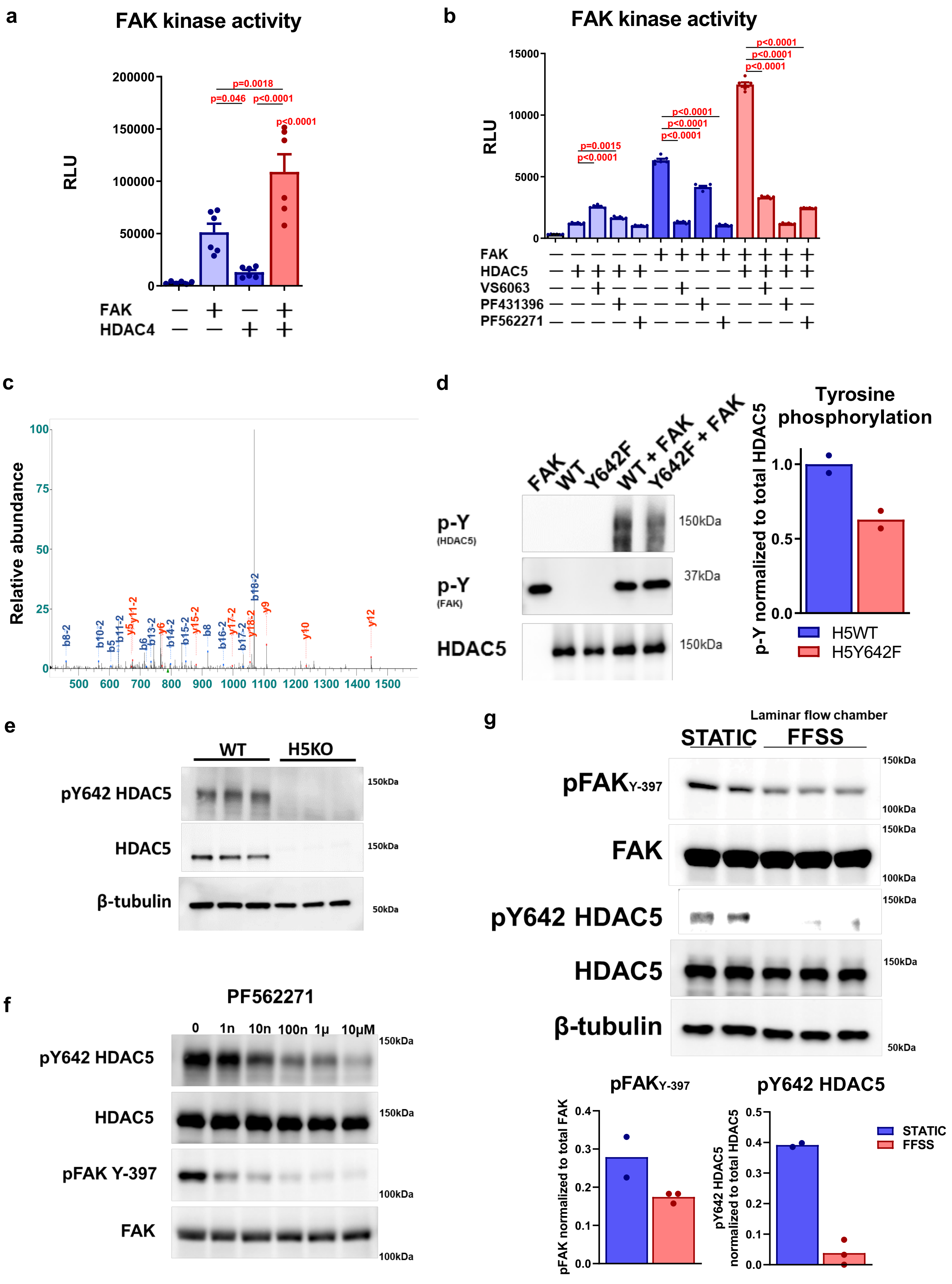

Supplemental Figure 7

(a) Recombinant HDAC4 was subjected to bioluminescence-based kinase assays as in Figure 5D. Increased FAK phosphorylation activity is noted when HDAC4 is added as a substrate. N=6/group. (b) FAK inhibitors reduced FAK kinase activity towards HDAC5, N=6/group. (c) MS/MS fragmentation spectrum of the phosphorylated peptide KKLFSDAQPLQPLQVY#QAPL produced from the chymotrypsin digestion of HDAC5 in the presence of FAK treatment (#indicates site of phosphorylation). A confident assignment of the phosphate to the tyrosine was demonstrated (score = 35.0) with the site assignment program Ascore. The extracted ion chromatograms are shown in Figure 6 a,b. (d) 293T cells were transfected with FLAG-tagged WT or Y642F HDAC5. 48 hours later, FLAG immunoprecipitation was performed, followed by elution with excess amounts of FLAG peptide. Eluted HDAC5 protein was then subjected to kinase assay with recombinant FAK, followed by immunoblotting as indicated. FAK-dependent HDAC5 tyrosine phosphorylation was significantly reduced in the Y642F mutant. N=2. (e) Control (WT) and HDAC5 mutant Ocy454 cell lysates were subjected to immunoblotting as indicated. HDAC5 Y642 phosphorylation was not detected in HDAC5 deficient cells. (f) Ocy454 cells were treated with FAK inhibitors at the indicated doses for 60 minutes followed by immunoblotting as indicated. FAK inhibitors dose-dependently reduced HDAC5 Y642 phosphorylation. (g) Ocy454 cells were grown on collagen coated slides and placed in flow chambers for 45 minutes plus/minus laminar flow at 8 dynes/cm2. Immediately thereafter, cells were collected for immunoblotting as indicated. N=2, STATIC. N=3, FFSS. One-way ANOVA followed by Tukey-Kramer post hoc test (a, b) was used. p values adjusted for multiple comparisons are shown (a, b). Data are expressed as mean ± SEM (a, b) and mean with individual values (d, g in the bottom panel). Source data are provided as a Source Data file.

Supplemental Figure 8 (related to Figure 7)

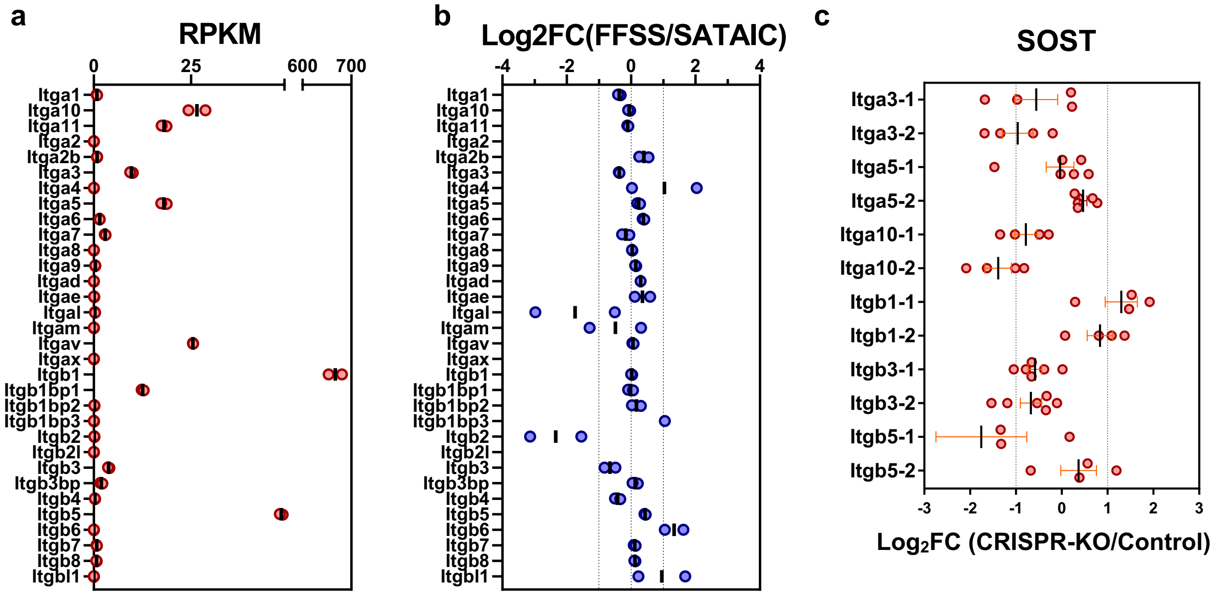

Supplemental Figure 8

(a) Expression of integrin isoforms was interrogated in Ocy454 cell RNA-seq data (24). N=2. (b) Effects of FFSS exposure on integrin isoform expression. N=2. (c) Bulk cell populations were generated in which the indicated integrin isoform was targeted via CRISPR/Cas9. Basal Sost expression was then measured by RT-qPCR. None of the perturbations tested dramatically altered basal Sost expression. N=4 (Itga3-1, Itga3-2, Itga5-1, Itga5-2, Itga10-1, Itga10-2, Itgb1-1, Itgb1-2, Itgb3-1, Itgb3-2, Itgb5-1, and Itgb5-2). N=6 (Itga5-1, Itga5-2, Itga10-1, Itga10-2, Itgb1-1, Itgb1-2, Itgb3-1, and Itgb3-2). One-way ANOVA followed by Tukey-Kramer post hoc test (c) was used. Data are expressed as mean ± SEM (c) and mean with individual values (a, b). Source data are provided as a Source Data file.

References

1. Prasad J, Wiater BP, Nork SE, Bain SD, and Gross TS. Characterizing gait induced normal strains in a murine tibia cortical bone defect model. Journal of biomechanics. 2010;43(14):2765-70.
2. Srinivasan S, Balsiger D, Huber P, Ausk BJ, Bain SD, Gardiner EM, et al. Static Preload Inhibits Loading-Induced Bone Formation. JBMR Plus. 2019;3(5):e10087.
3. Wein MN, Liang Y, Goransson O, Sundberg TB, Wang J, Williams EA, et al. SIKs control osteocyte responses to parathyroid hormone. Nature communications. 2016;7:13176.
