# Supplementary material for: A FAK/HDAC5 signaling axis controls osteocyte mechanotransduction
Source: Nat Commun. 2020 Jul 1;11:3282. doi: 10.1038/s41467-020-17099-3 (PMC7329900; doi:10.1038/s41467-020-17099-3)
Supplement: Supplementary file 3 — Description of Additional Supplementary Files [file 41467_2020_17099_MOESM3_ESM.pdf]

Description of Additional Supplementary Files

File Name: Supplementary Data 1
Description: RNA-seq datasets

File Name: Supplementary Data 2
Description: Oligonucleotide sequences used

File Name: Supplementary Data 3
Description: Quality control metrics for RNA-seq libraries

File Name: Supplementary Data 4
Description: Primary LS-MS/MS data, related to Figure 6a-b and Supplemental Figure 7c.
