# Supplementary material for: A FAK/HDAC5 signaling axis controls osteocyte mechanotransduction
Source: Nat Commun. 2020 Jul 1;11:3282. doi: 10.1038/s41467-020-17099-3 (PMC7329900; doi:10.1038/s41467-020-17099-3)
Supplement: Supplementary file 8 — Reporting Summary [file 41467_2020_17099_MOESM8_ESM.pdf]

nature research

Corresponding author(s): Marc N Wein

Last updated by author(s): Apr 28, 2020

Reporting Summary

Nature Research wishes to improve the reproducibility of the work that we publish. This form provides structure for consistency and transparency in reporting. For further information on Nature Research policies, seeAuthors & Referees and theEditorial Policy Checklist .

Statistics

For all statistical analyses, confirm that the following items are present in the figure legend, table legend, main text, or Methods section.

n/a	Confirmed	
☐	☒	The exact sample size (*n*) for each experimental group/condition, given as a discrete number and unit of measurement
☐	☒	A statement on whether measurements were taken from distinct samples or whether the same sample was measured repeatedly
☐	☒	The statistical test(s) used AND whether they are one- or two-sided<br>*Only common tests should be described solely by name; describe more complex techniques in the Methods section.*
☐	☒	A description of all covariates tested
☐	☒	A description of any assumptions or corrections, such as tests of normality and adjustment for multiple comparisons
☐	☒	A full description of the statistical parameters including central tendency (e.g. means) or other basic estimates (e.g. regression coefficient) AND variation (e.g. standard deviation) or associated estimates of uncertainty (e.g. confidence intervals)
☐	☒	For null hypothesis testing, the test statistic (e.g. *F*, *t*, *r*) with confidence intervals, effect sizes, degrees of freedom and *P* value noted<br>*Give P values as exact values whenever suitable.*
☒	☐	For Bayesian analysis, information on the choice of priors and Markov chain Monte Carlo settings
☒	☐	For hierarchical and complex designs, identification of the appropriate level for tests and full reporting of outcomes
☐	☒	Estimates of effect sizes (e.g. Cohen's *d*, Pearson's *r*), indicating how they were calculated

Our web collection on statistics for biologists contains articles on many of the points above.

Software and code

Policy information about availability of computer code

Data collection	Windows (ver 10), Microsoft Excel for Office 365 (ver 2016), StepOne Software (ver 2.3), Zeiss Zen software (ver 2.6), Epson scan (ver 3.9.4.7US), Azure biosystems cSeries capture software (ver 1.9.7.0802)
Data analysis	Windows (ver 10), Microsoft Excel for Office 365 (ver 2016), Microsoft Word for Office 365 (ver 2016), GraphPad Prism (ver 8.4.2), StepOne Software (v2.3), NIH ImageJ (ver 1.52a), EgdeR package (ver 3.24.1), Enrichr (https://amp.pharm.mssm.edu/Enrichr/) (ver January 7th, 2020), GeneXplain (ver 4.8), ingenuity pathway analysis (Build version: 430520M Content version: 31813283), Overlap stats (nemates.org/MA/progs/overlap_stats.html) (ver 2019) , Sequest (SRF v.5), HTSeq (ver 0.9.1)

For manuscripts utilizing custom algorithms or software that are central to the research but not yet described in published literature, software must be made available to editors/reviewers. We strongly encourage code deposition in a community repository (e.g. GitHub). See the Nature Research guidelines for submitting code & software for further information.

Data

Policy information about availability of data

All manuscripts must include a data availability statement. This statement should provide the following information, where applicable:

- Accession codes, unique identifiers, or web links for publicly available datasets
- A list of figures that have associated raw data
- A description of any restrictions on data availability

All data generated or analyzed during this study are included in this published article (and its supplementary information files). The RNA-seq data has been deposited to GEO under accession numbers GSE139604 and GSE144265. Source data underlying all figures are provided as a Source Data File.

Field-specific reporting

Please select the one below that is the best fit for your research. If you are not sure, read the appropriate sections before making your selection.

[x] Life sciences [ ] Behavioural & social sciences [ ] Ecological, evolutionary & environmental sciences

For a reference copy of the document with all sections, see nature.com/documents/nr-reporting-summary-flat.pdf

Life sciences study design

All studies must disclose on these points even when the disclosure is negative.

Sample size	Power calculations were performed based on pilot experiments.
Data exclusions	No data were excluded from the analyses.
Replication	Each experiment was repeated at least three times. We confirm that all attempts at replication were successful.
Randomization	All animals were randomly divided to the control and experimental groups. The samples and cells are randomized into different groups before treatment.
Blinding	All animal bone analyses, group allocation and data collection were performed on a blinded basis.

Reporting for specific materials, systems and methods

We require information from authors about some types of materials, experimental systems and methods used in many studies. Here, indicate whether each material, system or method listed is relevant to your study. If you are not sure if a list item applies to your research, read the appropriate section before selecting a response.

Materials & experimental systems

n/a	Involved in the study	
[ ]	[x]	Antibodies
[ ]	[x]	Eukaryotic cell lines
[x]	[ ]	Palaeontology
[ ]	[x]	Animals and other organisms
[x]	[ ]	Human research participants
[x]	[ ]	Clinical data

Methods

n/a	Involved in the study	
[x]	[ ]	ChIP-seq
[x]	[ ]	Flow cytometry
[x]	[ ]	MRI-based neuroimaging

Antibodies

Antibodies used	For immunoblotting, FAK (1:1000, Cell Signaling Technology, 13009), p-FAK(Y-397) (1:500, Cell Signaling Technology, 8556), phospho-Paxillin (1:250, Cell Signaling Technology, 2541), Paxillin (1:500, Cell Signaling Technology, 12065), HDAC4 (1:1000, Abcam, ab12172), phospho-HDAC4/5/7 (S246/S259/S155) (1:250, Cell Signaling Technology, 3443), DYKDDDDK tag (1:1000, Cell Signaling Technology, 2368), p-p44/42 MAPK (T202/Y204) (pERK) (1:500, Cell Signaling Technology, 9101), p44/42 MAPK (Erk1/2) (1:1000, Cell Signaling Technology, 9102), p-Y-1000 (1:1000, Cell Signaling Technology, 8954), beta-tubulin (1:250, Cell Signaling Technology, 5346), The HDAC5 pY642 antibody (1:1000) was produced using YenZym Antibodies' P-site™ Antibody Service protocol.<br>For immunohistochemistry and immunocytochemistry, mSOST biotinylated (1:50, R&D systems, BAF1589), non-phospho (active) β-catenin (Ser33/37/Thr41) (1:50, Cell Signaling Technology, 8814), HDAC5 (C-11) (1:50, Santa Cruz Biotechnology, sc-133225), HDAC4 (1:50, Abcam, ab12174), FLAG (1:50, SIGMA, F1804), donkey anti-mout IgG with Alexa 488 (1:200 Life Technologies, A-21202), donkey anti-rabbit IgG with Alexa 568 (1:200 Life Technologies, A10042).
Validation	Validation statements of Commercial available antibodies are on the manufacturer's website.<br>The validation of the HDAC5 pY642 antibody was provided in the manuscript (Figure 6C-F and Supplemental Figure 7E-G).<br>FAK (Cell Signaling Technology, 13009), https://www.cellsignal.com/products/primary-antibodies/fak-d2r2e-rabbit-mab/13009<br>p-FAK(Y-397) (Cell Signaling Technology, 8556), https://www.cellsignal.com/products/primary-antibodies/phospho-fak-tyr397-d20b1-rabbit-mab/8556<br>phospho-Paxillin (Cell Signaling Technology, 2541), https://www.cellsignal.com/products/primary-antibodies/phospho-paxillin-tyr118-antibody/2541<br>Paxillin (Cell Signaling Technology, 12065), https://www.cellsignal.com/products/primary-antibodies/paxillin-d9g12-rabbit-mab/12065<br>HDAC4 (Abcam, ab12172), https://www.abcam.com/hdac4-antibody-ab12172.html<br>phospho-HDAC4/5/7 (S246/S259/S155) (Cell Signaling Technology, 3443), https://www.cellsignal.com/products/primary-

antibodies/phospho-hdac4-ser246-hdac5-ser259-hdac7-ser155-d27b5-rabbit-mab/3443
DYKDDDDK tag (Cell Signaling Technology, 2368), https://www.cellsignal.com/products/primary-antibodies/dykddddk-tag-antibody-binds-to-same-epitope-as-sigma-s-anti-flag-m2-antibody/2368
p-p44/42 MAPK (T202/Y204) (pERK) (Cell Signaling Technology, 9101), https://www.cellsignal.com/products/primary-antibodies/phospho-p44-42-mapk-erk1-2-thr202-tyr204-antibody/9101
p44/42 MAPK (Erk1/2) (Cell Signaling Technology, 9102), https://www.cellsignal.com/products/primary-antibodies/p44-42-mapk-erk1-2-antibody/9102
p-Y-1000 (Cell Signaling Technology, 8954), https://www.cellsignal.com/products/primary-antibodies/phospho-tyrosine-p-tyr-1000-multimab-rabbit-mab-mix/8954
beta-tubulin (Cell Signaling Technology, 5346), https://www.cellsignal.com/products/antibody-conjugates/b-tubulin-9f3-rabbit-mab-hrp-conjugate/5346
mSOST biotinylated (R&D systems, BAF1589), https://www.rndsystems.com/products/mouse-sost-sclerostin-biotinylated-antibody_baf1589
non-phospho (active) β-catenin antibody (Cell Signaling Technology, 8814), https://www.cellsignal.com/products/primary-antibodies/non-phospho-active-b-catenin-ser33-37-thr41-d13a1-rabbit-mab/8814
HDAC4 (Abcam, ab12174), https://www.abcam.com/hdac4-antibody-ab12172.html
FLAG (SIGMA, F1804), https://www.sigmaaldrich.com/catalog/product/sigma/f1804?lang=en&region=US
donkey anti-mout IgG with Alexa 488 (Life Technologies, A-21202), https://www.thermofisher.com/antibody/product/Donkey-anti-Mouse-IgG-H-L-Highly-Cross-Adsorbed-Secondary-Antibody-Polyclonal/A-21202
donkey anti-rabbit IgG with Alexa 568 (Life Technologies, A10042), https://www.thermofisher.com/antibody/product/Donkey-anti-Rabbit-IgG-H-L-Highly-Cross-Adsorbed-Secondary-Antibody-Polyclonal/A10042

Eukaryotic cell lines

Policy information about cell lines

Cell line source(s)

Ocy454 cells were obtained from Center for Skeletal Research at Massachuestts General Hospital (Ocy454 cells were isolated from the long bones of 4-wk-old SV40TAg and 8KbDmp1–green fluorescent protein (GFP) double-transgenic mice. Ref J. Biol. Chem. 290, 16744–16758). 293T and Saos-2 cells were obtained from ATCC.

Authentication

We validated Ocy454 cells previously (The journal of the American Society for Bone and Mineral Research, 2015;30 (3):400-11). The authentication of Ocy454 were routinely tested by Sost and Dmp1 expression at 37°C and examined for osteocytic morphology (ß-actin primers; 5'-CCTCTATGCCAACACAGTGC-3' and 5'-ACATCTGCTGGAAGGTGGAC-3', SOST; 5'-GCCTCATCTGCCTACTTGTG-3' and 5'-CTGTGGCATCATTCCTGAAG-3', DMP-1; 5'-AGGACTCCACAGACACCACA-3' and 5'-GGGTATCTTGGGCACTGTTT-3'). 293T (ATCC CRL-3216) and Saos-2 (ATCC HTB-85) are authenticated by ATCC.

Mycoplasma contamination

We tested and confirmed that all cell lines were mycoplasma negative.

Commonly misidentified lines
(See ICLAC register)

No commonly misidentified lines were used.

Animals and other organisms

Policy information about studies involving animals; ARRIVE guidelines recommended for reporting animal research

Laboratory animals

The following published genetically-modified strains were used: Hdac4 floxed mice (RRID: MGI:4418117), germline Hdac5-deficient mice (RRID: MGI:3056065), and DMP1-Cre mice (RRID: MGI:3784520). Mice were backcrossed to C57BL/6 (Jacson laboratory) mice for at least seven generations. Floxed littermates without Cre were used as wild-type controls. Genotypes were determined by PCR using primers described in Supplemental Table 2. 20 week old wild type, HDAC4 conditional knockout (cKO, HDAC4 f/f;DMP1-Cre), HDAC5 knockout, and double knockout (DKO, HDAC4 f/f;HDAC5-/-;DMP1-Cre) female mice were subjected to 3 (for acute) or 9 (for long-term) loading. 6-7 weeks old female mice were treated with vehicle or FAK inhibitor. All mice were housed under the conditions of 21.9 ± 0.8°c, 45 ± 15% humidity, and 12-hour light/dark cycle (7 am-7 pm light/7 pm-7 am dark).

Wild animals

The study did not involve wild animals.

Field-collected samples

The study did not involve samples collected from the field.

Ethics oversight

All animals were housed in the Center for Comparative Medicine at the Massachusetts General Hospital and all experiments were approved by the hospital's Subcommittee on Research Animal Care. Power calculations were performed based on pilot experiments.

Note that full information on the approval of the study protocol must also be provided in the manuscript.
